# Moraxella osloensis Bacteremia Presenting as Abdominal Pain Following Dental Surgery

**DOI:** 10.7759/cureus.80663

**Published:** 2025-03-16

**Authors:** Andrew Holzman, Brennan Hand, Wayne A Martini, Douglas Rappaport

**Affiliations:** 1 Emergency Medicine, Mayo Clinic Alix School of Medicine, Phoenix, USA; 2 Emergency Medicine, Mayo Clinic, Phoenix, USA

**Keywords:** abdominal pain, bacteremia, dental surgery, moraxella osloensis, m. osloensis

## Abstract

We report a case of *Moraxella osloensis* bacteremia in a 66-year-old male presenting with acute abdominal pain and intermittent low-grade fevers following a dental extraction. The patient was diagnosed on return to the emergency department after cultures drawn during an initial visit produced a positive result for the organism on polymerase chain reaction (PCR). He was discharged without complication following admission to the hospital for intravenous antibiotics. Clinically significant *M. osloensis* infection is rare, and we have not found other reports describing a similar presentation with this organism.

## Introduction

*Moraxella osloensis* is a gram-negative, non-fermentative, oxidase-positive coccobacillus [1] which is a genitourinary tract and upper respiratory commensal in humans, although it has been implicated in a variety of infectious processes [2]. *M. osloensis* virulence and pathogenicity are related to heat-stable endotoxin [3]. It is one of six species in the *Moraxella* genus with clinical relevance, which also include *M. catarrhalis *(otitis media, sinusitis, lower respiratory infection) [4], *M. lacunata* (conjunctivitis) [2], *M. nonliquefaciens *(secondary respiratory infections) [2],* M. lincolnii *and *M. atlantae* [5]. While *Moraxella* species are frequently found in the oropharynx, *M. osloensis’ *morphology and gram-negative pattern mean it can be confused with *Neisseria gonorrhoeae* when sampled in the genitourinary tract [2].

Clinically significant infections with *M. osloensis* are considered extremely rare, with a limited number of cases reported stretching back to the taxonomic identification of the species in the early 1970s [5]. These have frequently included isolation from monoarticular septic joints, which may compound diagnostic confusion with *N. gonorrhoeae*. Presentation has also included meningitis [6], brain gliomatosis [7], and catheter-associated infection [8].

Differentiation of *M. osloensis* from other species requires sequencing of the 16s ribosomal gene or matrix-assisted laser desorption/ionization time-of-flight (MALDI-TOF) spectrometry, which may be cumbersome and unavailable in some clinical microbiology laboratories [5].

In many patients, culture of *M. osloensis* from the peripheral blood may be clinically insignificant, with one study finding only two of nine patients with positive culture presenting clinically relevant symptoms [5]. This rate may be higher in chronically ill populations - an investigation of *M. osloensis* catheter and blood infection in cancer patients receiving chemotherapy identified 10 infections, with patients experiencing fever and neutropenia [9].

In general, the prognosis even of clinically significant infection is good, with susceptibility to cell-wall active antibiotics such as ampicillin and ceftriaxone reported [5]. A fatality was described in a lung cancer patient with underlying immunosuppression [10].

Despite the rarity of this organism as a human pathogen, it has been identified in a variety of clinical presentations. Awareness of *M. osloensis* and the *Moraxella* genus as an element of the differential for these conditions is important for physicians in the emergency department. Prompt recognition of this species as a potential pathogen will allow leverage of its high degree of susceptibility to treatment and avoidance of complications in chronically ill or immunocompromised patients.

## Case presentation

A 66-year-old male with a past medical history of prostate cancer and benign prostatic hyperplasia treated with laser enucleation in 2021, gastroesophageal reflux, and a recent diagnosis of *H. pylori*-negative gastritis secondary to chronic non-steroidal anti-inflammatory drug (NSAID) use presented to the emergency department with abdominal pain and intermittent low-grade fevers of one week’s duration following a dental extraction. The patient reported the pain was located in the epigastrium with radiation to the right upper quadrant. The patient’s dental extraction was performed with prophylactic amoxicillin prior to and following the procedure.

In the emergency department, the patient was hypotensive with a blood pressure of 90/43 (which was around the baseline blood pressure for this patient) with a heart rate of 81 bpm. He was afebrile at 36.6 degrees Celsius and saturating at 100% on room air with a respiratory rate of 16. The abdomen was mildly tender to palpation in the epigastric region without guarding; there was no distension and bowel sounds were normal. The oropharynx was clear with normal mucous membranes. Abdominal CT was unremarkable for acute processes. Laboratory evaluations are summarized in Table 1. 

**Table 1:** Complete blood count (CBC), basic metabolic panel (BMP), and lactate values MCV: mean corpuscular volume, RBC: red blood cells, WBC: white blood cells

Item	Value	Normal Range (adult male)
CBC		
Hemoglobin	12.8	13.5–17.5 g/dL
Hematocrit	37.5	41–50%
Erythrocytes	4.26	4.7–6.1 x10⁶/µL
MCV	88.0	80–100 fL
RBC Distribution Width	12.3	11.5–14.5%
Platelet Count	178	150–450 x10³/µL
Leucocytes	4.4	4.5–11.0 x10³/µL
Differential		
Neutrophils	3.18	2.0–7.0 x10³/µL
Lymphocytes	0.81	1.0–3.0 x10³/µL
Monocytes	0.34	0.2–0.8 x10³/µL
Eosinophils	0.08	0.02–0.5 x10³/µL
Basophils	0.03	0 –0.3 x10³/µL
Nucleated RBCs	0.0	0/100 WBC
BMP		
Sodium	135	135–145 mEq/L
Potassium	4.1	3.5–5.0 mEq/L
Chloride	101	98–107 mEq/L
Bicarbonate	24	22–29 mEq/L
Anion Gap	10	8–16 mEq/L
Blood Urea Nitrogen	23.1	6–20 mg/dL
Creatinine	1.10	0.74–1.35 mg/dL
Lactate	0.8	0.5–2.2 mmol/L

Complete blood count, basic metabolic panel, hepatic function panel, lipase and urinalysis were normal, and blood cultures were sent but not returned at the time of initial evaluation.

The patient’s abdominal pain was attributed to gastritis potentially related to use of NSAIDs, and he was discharged with instructions to follow up with a gastroenterologist.

One week following this initial presentation, the patient returned to the emergency department after blood cultures returned gram-negative cocci. Low-grade fevers, fatigue and headaches had persisted but the patient continued to deny upper respiratory or urinary symptoms. At 49 hours, *M. osloensis* was detected by polymerase chain reaction (PCR). The patient was admitted to the hospital and started on a course of intravenous ceftriaxone. Three days later, he was discharged to home health with a peripherally inserted central catheter (PICC) for completion of the two-week course with resolution of his symptoms and he experienced no subsequent complications.

## Discussion

This case contributes to the growing basis of literature showing *Moraxella osloensis* as a pathogen capable of causing systemic infection in an immunocompetent patient without severe chronic disease. While *M. osloensis* is traditionally considered a commensal organism of human skin and mucosa, emerging reports, including this case, highlight its potential to cause serious infections. As discussed, existing literature suggests such infections are rare, and we have not identified prior reports linking *M. osloensis* to bacteremia following a dental procedure. 

Systemic infection due to oral flora is a recognized risk following dental procedures, with infectious endocarditis being a major concern. The highest risk of bacteremia is associated with dental extractions, though even routine interventions such as professional cleanings and daily tooth brushing may introduce bacteria into the bloodstream [11]. Documented cases of bacteremia following dental work include *Streptococcus intermedius* leading to liver abscess after a dental cleaning [12] and *Pseudomonas aeruginosa* causing mycotic aneurysm of the carotid following molar extraction [13]. While direct causation of bacteremia by dental work cannot be definitively established in this case, the temporal association is notable. 

*Moraxella osloensis* is a gram-negative, aerobic, non-motile, oxidase-positive coccobacillus that was reclassified as a separate species in 1967, having previously been grouped with *Moraxella nonliquefaciens*. It has been isolated from various environmental sources, including sinks, laundry, and anesthetic agents. While historically reported in immunocompromised patients, cases in immunocompetent individuals have been documented, including those involving bacteremia, endocarditis, meningitis, osteomyelitis, pneumonia, and peritonitis in peritoneal dialysis patients. Its role in peritonitis suggests that *M. osloensis* may have the potential for mucosal invasion, a plausible explanation for the abdominal pain observed in our patient. 

In this case, we attribute the patient's primary presenting symptom of acute abdominal pain to bacteremia, potentially due to gastrointestinal mucosal invasion by *M. osloensis*. While the pain could alternatively be explained by an exacerbation of chronic gastritis related to NSAID use, bacteremia has been associated with acute abdominal pain, particularly in infections of gastrointestinal origin, as seen in *Brucella* bacteremia [14] and *Yersinia pseudotuberculosis* (pseudoappendicitis) [15]. Although *M. osloensis* is not a typical gastrointestinal pathogen, its involvement in peritonitis cases suggests a capacity for peritoneal infection [16,17]. 

The patient's favorable response to ceftriaxone aligns with prior reports of *M. osloensis* susceptibility to cephalosporins. While dental prophylaxis with amoxicillin did not prevent infection in this case, this may not necessarily indicate resistance, given the variable effectiveness of amoxicillin prophylaxis in preventing bacteremia post-dental procedures [11]. 

Interestingly, despite presenting without an elevated white blood cell count, this is not uncommon in bacteremia, with studies reporting that up to 52% of bacteremic patients may have a normal leukocyte count [18]. This case adds to the literature by demonstrating that *M. osloensis* can be a clinically relevant pathogen in previously healthy individuals and reinforces the importance of considering this organism in post-dental procedure infections [19]. 

Advancements in microbiological identification techniques, such as 16S ribosomal RNA sequencing and MALDI-TOF mass spectrometry, have improved recognition of *M. osloensis* infections [17,19,20]. Given the growing body of literature identifying *M. osloensis* as a pathogen in both immunocompromised and immunocompetent individuals, further research is warranted to elucidate the epidemiology, risk factors, and optimal management strategies for these infections. 

Overall, while definitive causation between the patient’s bacteremia and the recent dental procedure cannot be established, the clinical course strongly supports this as a probable association. The rapid onset of symptoms following dental surgery, coupled with the patient's positive response to antibiotics, suggests that *M. osloensis* was a likely source of bacteremia and subsequent acute symptoms. However, the inherent challenge in proving this association remains a limitation in fully understanding the implications of this case. 

## Conclusions

This case highlights the importance of surveillance for rare pathogens in patients presenting with infectious symptoms. Additionally, it proposes a possible presentation of infection with *M. osloensis*. Continued surveillance for rare pathogens is an important element of emergency department practice and its contribution to public health.

## References

[1] Shah SS, Ruth A, Coffin SE (2000). Infection due to Moraxella osloensis: case report and review of the literature. Clin Infect Dis.

[2] Cooke FJ, Slack MP (2017). Gram-negative coccobacilli. Infectious Diseases.

[3] Tan L, Grewal PS (2002). Endotoxin activity of Moraxella osloensis against the grey garden slug, Deroceras reticulatum. Appl Environ Microbiol.

[4] Verghese A, Berk SL (1991). Moraxella (Branhamella) catarrhalis. Infect Dis Clin North Am.

[5] Khalife M, Merashli M, Kanj SS (2019). Moraxella nonliquefaciens septic arthritis in a hematopoietic stem cell transplant patient a case report and review of the literature. J Infect Public Health.

[6] Fox-Lewis A, Coltart G, Rice S, Sen R, Gourtsoyannis Y, Hyare H, Gupta RK (2016). Extensive subclinical sinusitis leading to Moraxella osloensis meningitis. IDCases.

[7] Strojnik T, Kavalar R, Gornik-Kramberger K, Rupnik M, Robnik SL, Popovic M, Velnar T (2020). Latent brain infection with Moraxella osloensis as a possible cause of cerebral gliomatosis type 2: a case report. World J Clin Oncol.

[8] Hadano Y, Ito K, Suzuki J, Kawamura I, Kurai H, Ohkusu K (2012). Moraxella osloensis: an unusual cause of central venous catheter infection in a cancer patient. Int J Gen Med.

[9] Han XY, Tarrand JJ (2004). Moraxella osloensis blood and catheter infections during anticancer chemotherapy: clinical and microbiologic studies of 10 cases. Am J Clin Pathol.

[10] Lee MY, Kim MH, Lee WI, Kang SY (2021). Bacteremia caused by Moraxella osloensis: a fatal case of an immunocompromised patient and literature review. Clin Lab.

[11] Rutherford SJ, Glenny AM, Roberts G, Hooper L, Worthington HV (2022). Antibiotic prophylaxis for preventing bacterial endocarditis following dental procedures. Cochrane Database Syst Rev.

[12] Livingston LV, Perez-Colon E (2014). Streptococcus intermedius bacteremia and liver abscess following a routine dental cleaning. Case Rep Infect Dis.

[13] Knouse MC, Madeira RG, Celani VJ (2002). Pseudomonas aeruginosa causing a right carotid artery mycotic aneurysm after a dental extraction procedure. Mayo Clin Proc.

[14] Yılmaz Çelebi M, Böncüoğlu E, Kıymet E (2024). Comparative analysis of pediatric brucellosis cases with and without bacteremia. Vector Borne Zoonotic Dis.

[15] Jones MW, Godana I, Hoilat GJ, Deppen JG (2025). Pseudoappendicitis. StatPearls [Internet].

[16] Yamada A, Kasahara K, Ogawa Y (2019). Peritonitis due to Moraxella osloensis: a case report and literature review. J Infect Chemother.

[17] Adapa S, Gumaste P, Konala VM, Agrawal N, Garcha AS, Dhingra H (2018). Peritonitis due to Moraxella osloensis: an emerging pathogen. Case Rep Nephrol.

[18] Seigel TA, Cocchi MN, Salciccioli J, Shapiro NI, Howell M, Tang A, Donnino MW (2012). Inadequacy of temperature and white blood cell count in predicting bacteremia in patients with suspected infection. J Emerg Med.

[19] Alkhatib NJ, Younis MH, Alobaidi AS, Shaath NM (2017). An unusual osteomyelitis caused by Moraxella osloensis: a case report. Int J Surg Case Rep.

[20] Li Y, Wang GQ, Ma XL, Li YB (2024). A rare case of acute meningitis caused by Moraxella osloensis. CNS Neurosci Ther.

